# Using Soft Gold

**Published:** 1898-09

**Authors:** Benj. Lord

**Affiliations:** New York.


ARTICLE V.

### USING SOFT GOLD.

DR. BENJ. LORD, NEW YORK.

I prefer soft foil because of its easy adaptation to the walls of the cavity and the greater ease and certainty with which it can be placed and packed without danger of tipping from becoming hard too soon, which is often true of cohesive gold.

Cohesive foils and other preparations of cohesive gold have their uses, such as the restoration of broken corners or the sides of the teeth, where it is desirable to to make such restorations, but I may say I think it is done quite too frequently, particularly with the front teeth, thereby giving them a more unsightly appearance than the marred or broken corners would present.

My experience and observation lead me to believe that cohesive gold is not so well adapted for general use as soft gold.

In my judgment the best form in which to use gold is in sheets, parts of the sheet being folded in strips of suitable widths for the cavity in which they are to be used. Strips or folds can be handled with more ease and certainty in process of filling than can rolls, pellets, or blocks.

It is my belief that too many forms of gold are prepared for our use, often leading to confusion and embarrassment, particularly on the part of the inexperienced. If my memory serves me correctly, the late Mr. R. S. Williams advertised that he prepared and had for sale one hundred and fifty different varieties of gold for dentists' use.

Filling teeth has so many difficulties to be overcome, and so much accuracy and fidelity are required, that we must follow certain lines for a long while, and, in the use of particular forms of gold, to arrive at excellence.

By using suitable points all cavities having three walls can be filled and the fillings contoured by the use of soft foil; this may be done with equal certainty and success with tinfoil or with a combination of gold and tin.

I depend mostly on a single form of instruments for placing the gold and for condensing it toward the walls of the cavity. Occasionally I use a point somewhat more curved to carry the gold already placed, with more force against the wall or into a corner of the cavity (always guarding against too much pressure), so that each additional layer may not only be placed against that which is already in position, but will be carried into it, thereby uniting the whole mass.

Then I come to the surface of condensing, which I begin with a small four sided instrument, curved to an angle of forty five degrees and pointed, the sides grooved, and the edges of the grooves serrated.

I assume, to insure the best results, that the foil has been accurately placed, the layers added in regular order, and condensed as solidly as possible by lateral condensing, and that the gold is raised sufficiently above the margins of the cavity, so that after the surface is made solid by using the point of the instrument and the edges of the groove, the filling will still have a contour or oval surface, if an approximate filling. I wish to attach great value to this surface condensation with small points, as it spreads the filling to some extent toward the walls of the cavity, and ties the whole mass together more securely. I speak of leaving the surface of the filling contoured or oval, which is certainly most natural, and, if so, highly desirable, but there is interest in the old style of the flat surface, or even in the concave surface, as there is much more certainty that the gold will be made solid against the walls of the cavity, with less care, when the surface is thus worked down. We find that filling so finished have done excellent service.

But by using points and making the pressure near the margin of the filling, so as to condense against the walls, and also using the edge of the groove of the instrument I have alluded to, full success may be secured, when the surface is left contoured.

I now come to the use of the broad instrument with one side flat and the other somewhat oval, both for further condensing and burnishing. The oval side will not be so likely to come in contact with the margins, and can be used to greater advantage to condense the surface; the flat side also possesses advantages over the oval for a part of the work.

I am now ready to give the margins their finish, and I think it will be seen that my instruments are well adapted for the purpose. They have one side flat and the other oval, are cut like burs, and are used much as a file of the same shape would be, For the final finishing I use a small instrument, the same as I use for the removal of tartar or any roughness about the teeth, and if there should still be any surplus gold at the margin, this instrument will be sure to detect and remove it.

I attach little if any value to the mere burnishing of the surface of fillings, only so far as it gives greater solidity to the surface. The great point in the finishing of fillings is the smoothness and solidity of the margins, so that no surplus material be left overhanging.

I do not think that any kind of a disk, sandpaper, or corundum should be used on the approximate surfaces for finishing fillings, as they are liable to cut away tooth-material or filling that ought not to be removed, and I do not like the use of the polishing strips, for the same reason. I also think that a bur or burnisher in the engine should never be used to reduce or polish fillings in the grinding surfaces of the teeth, as there is too much danger of reducing the cusps or injuring the margins of the cavity.

The grinding surface should be finished to correspond in irregularity to the natural surface of the tooth, and instruments may be used for that purpose. The burnishing is really of no importance except to give solidity.

I use Abbey's foil, and prefer No. 5. I cut a sheet generally into four pieces; if the cavity is small, I would cut the sheet in six pieces. I fold each piece into strips, for the larger cavities about an eighth of an inch wide, and for the smaller ones a sixteenth of an inch.

For the folding I use one side of my shears, instead of a table-knife or a paper-cutter, and I fold on a small cushion made of soft leather and filled with wool. I prefer this to anything harder. The strips are placed on a folded napkin, and I use the point of the instrument with which I am working to pick up the strip and carry it to the cavity. I usually cut the strips into two pieces, sometimes three. The instrument that I use and depend on mostly for placing, uniting, and condensing the folds is properly carved at the end, and an absolute point made by a very short bevel on three sides with a sharp file, and the fourth side touched at the point very slightly with the file, so that the line of the curve may not be changed.

I do not, as would be understood, condense with the point of the instrument, but with the side and edge of the bevel, and unite the folds with the point.

I strive as far as possible in placing the foil in the cavity, to hold the strip in so as to form a loop and bring the loop to the surface.—*International.*

